# Supplementary material for: Alteration of Gut Microbes in Benign Prostatic Hyperplasia Model and Finasteride Treatment Model
Source: Int J Mol Sci. 2023 Mar 21;24(6):5904. doi: 10.3390/ijms24065904 (PMC10057928; doi:10.3390/ijms24065904)
Supplement: Supplementary file 1 [file ijms-24-05904-s001.zip › ijms-2271987-supplementary.pdf]

Supplementary Materials

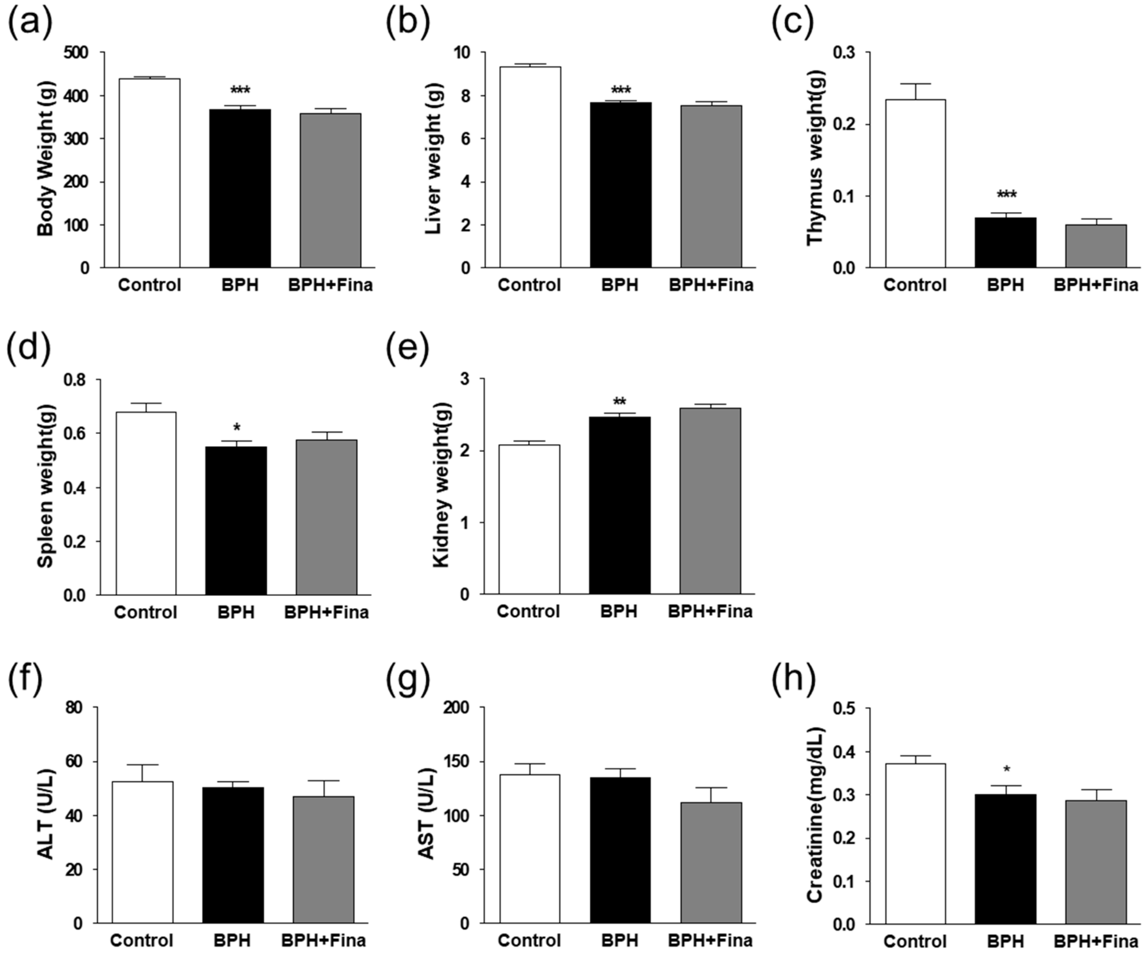

Figure S1 Body weight, Organ weight and biochemical analysis results

The change in body weight (a), organ weight (b–e), and liver and kidney function indicators were measured using a biochemical analyzer (f–h). Statistical analyses were performed using t-test and Mann–Whitney test. $^{*}p<0.05$, $^{**}p<0.01$, $^{***}p<0.001$ compared with control. Control group, injected with corn oil after sham operation; benign prostatic hyperplasia (BPH) group, injected with testosterone undecanoate (125 mg/kg) after castration; BPH+Fina group, injected with testosterone undecanoate (125 mg/kg) and finasteride (0.8 mg/kg) after castration.

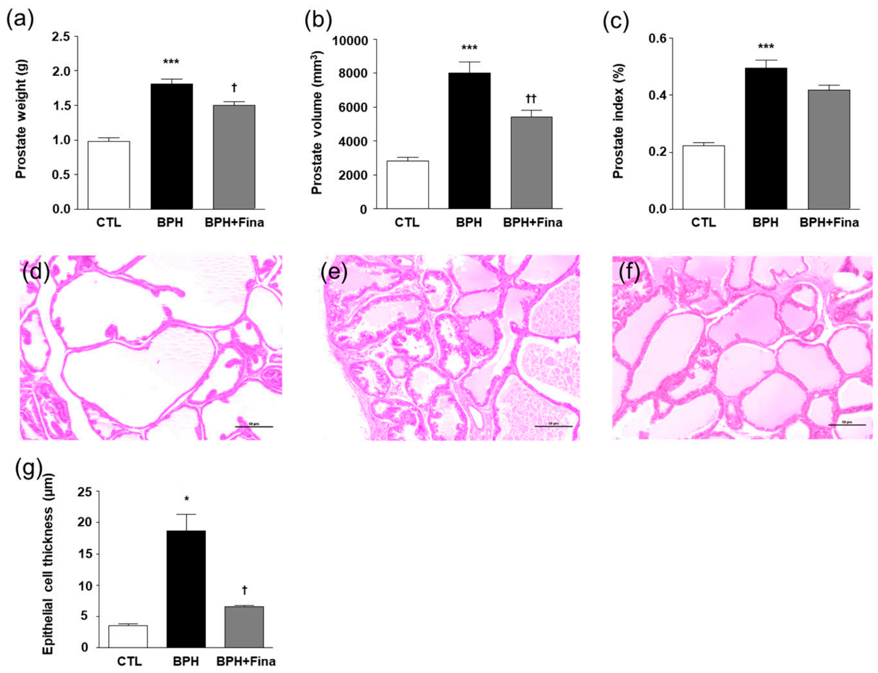

Figure S2 Change in prostate profiles

Prostate weight (a), prostate volume (b), and prostate index (c) were measured and calculated. Prostate volume was measured using a caliper. Prostate index was calculated using the formula: prostate weight/body weight *100. The microscopic images of prostate tissue stained with hematoxylin and eosin (H&E) are shown (80X magnification) (d–f) and measured epithelial thickness (g). Statistical analyses were performed using t-test and Mann–Whitney test. $^{*}p<0.05$, $^{***}p<0.001$ compared with control. $^{\dagger}p<0.05$, $^{\dagger\dagger}p<0.01$ compared with BPH. The groups: control, injected with corn oil following sham operation; BPH, injected as testosterone undecanoate (125 mg/kg) after castration; BPH+Fina, injected with testosterone undecanoate (125 mg/kg) and finasteride (0.8 mg/kg) after castration.

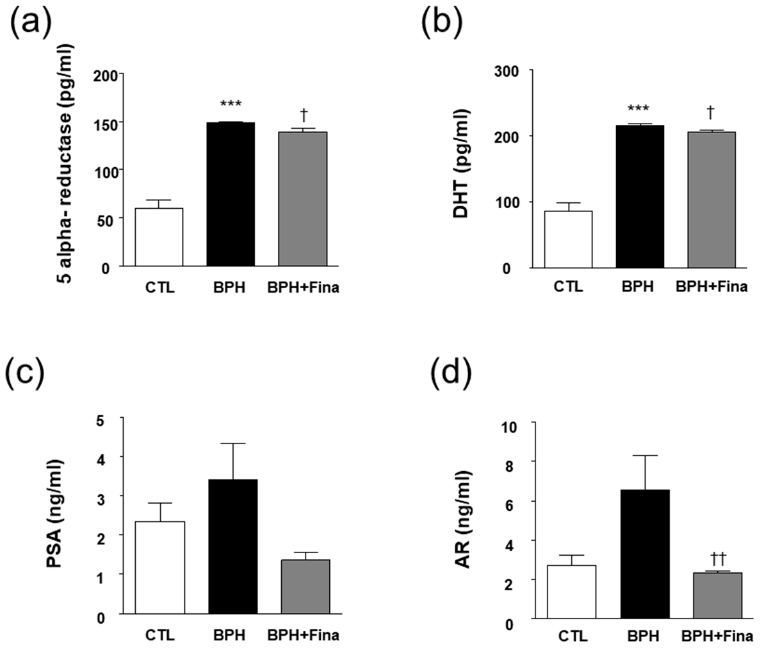

Figure S3 Change in BPH indicators

The levels of 5α-reductase (a) and dihydrotestosterone (DHT) (b) in serum and prostate-specific antigen (PSA) (c) and androgen receptor (AR) (d) in the prostate were measured using an ELISA kit. Statistical analyses were performed using t-test and Mann–Whitney test. $^{***}p<0.001$ compared with control. $^{\dagger}p<0.05$, $^{\dagger\dagger}p<0.01$ compared with BPH. The groups: control, injected with corn oil following sham operation; BPH, injected with testosterone undecanoate (125 mg/kg) after castration; BPH+Fina, injected with testosterone undecanoate (125 mg/kg) and finasteride (0.8 mg/kg) after castration.

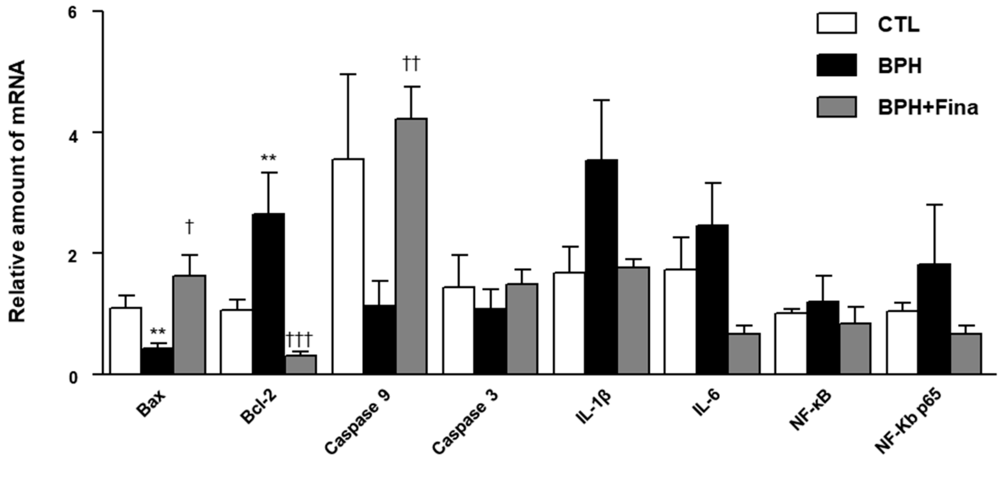

Figure S4 Changes in the transcriptome in prostate tissue

The transcriptomes of apoptosis markers (Bax, Bcl-2, capase-9, and capase-3) and inflammatory cytokines (IL-1β, IL-6, NF-κB, and NF-κB p65) in the prostate were measured using qPCR. Statistical analyses were performed using the t-test and Mann–Whitney test. $^{**}p<0.01$ compared with control. $^{\dagger}p<0.05$,$^{\dagger\dagger}p<0.01$, $^{\dagger\dagger\dagger}p<0.001$ compared with BPH. The groups: control, injected with corn oil following sham operation; BPH, injected with testosterone undecanoate (125 mg/kg) after castration; BPH+Fina, injected with testosterone undecanoate (125 mg/kg) and finasteride (0.8 mg/kg) after castration.

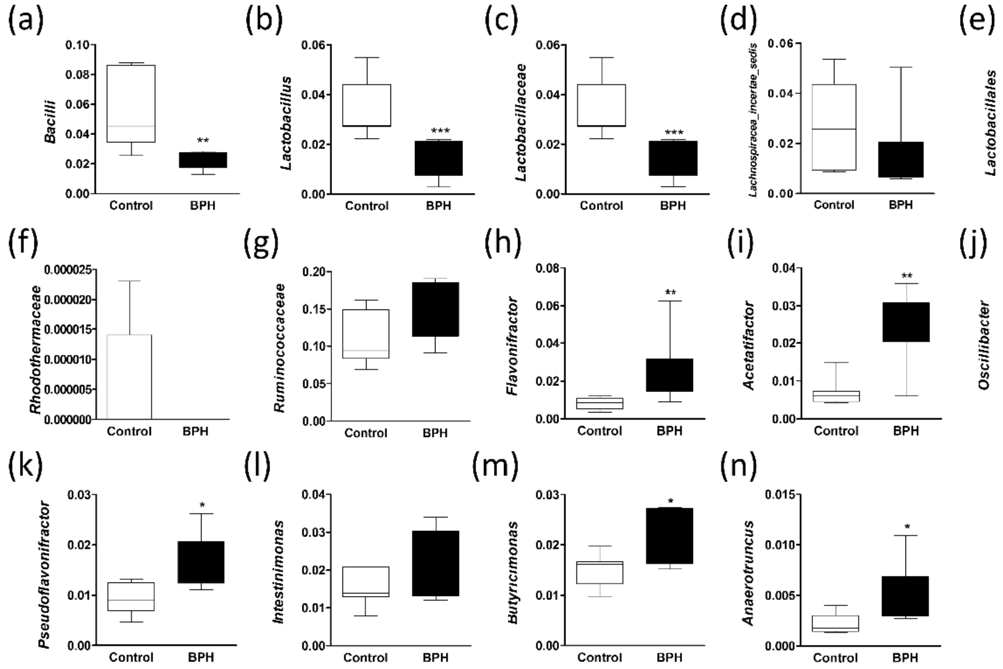

Figure S5 Alteration of relative bacterial abundance following BPH induction

Microbial abundance based on LEfSe method (b–o). Statistical analyses were performed using t-test and Mann–Whitney test. *p<0.05, **p<0.01, ***p<0.001. The groups: control, injected with corn oil following sham operation; BPH, injected with testosterone undecanoate (125 mg/kg) after castration.

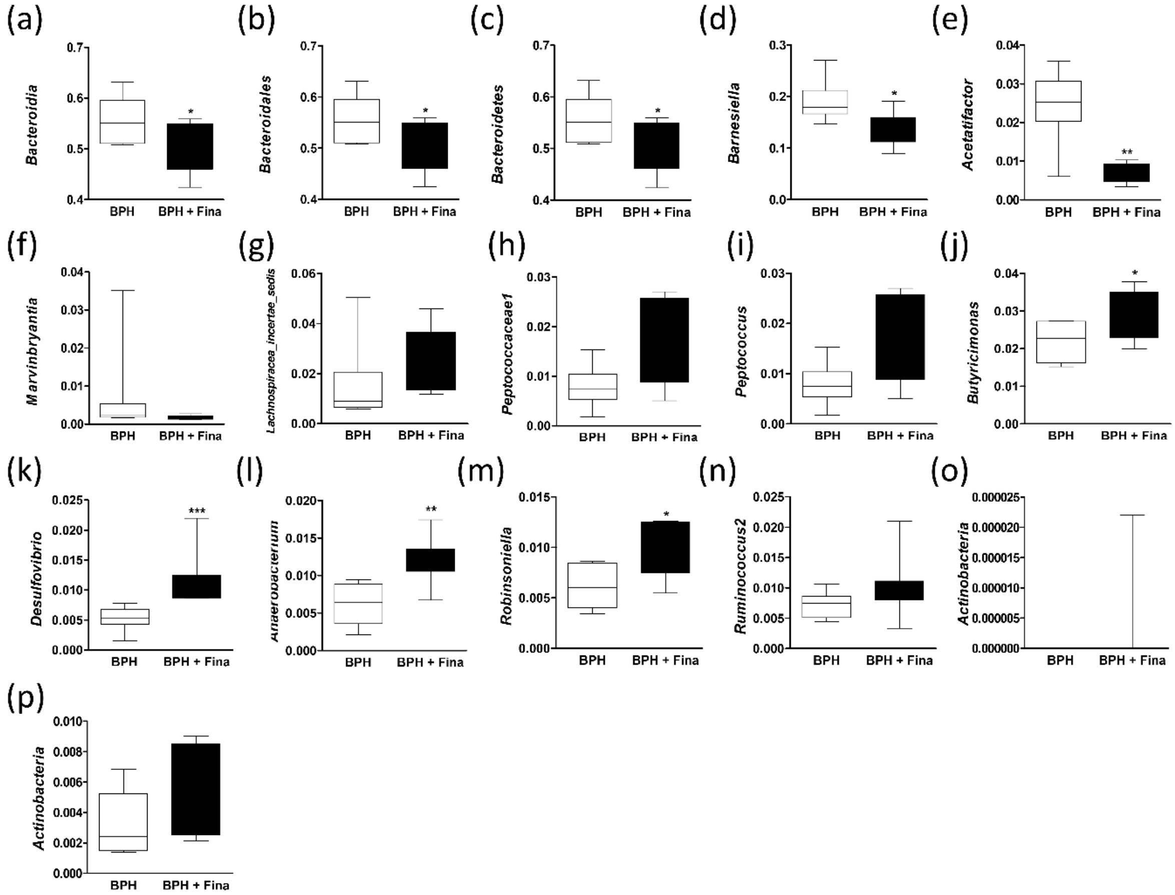

Figure S6 Alteration of relative bacterial abundance following finasteride treatment

Microbial abundance based on LEfSe method (b–o). Statistical analyses were performed using t-test and Mann–Whitney test. *p<0.05, **p<0.01, ***p<0.001. The groups: BPH, injected with testosterone undecanoate (125 mg/kg) after castration; BPH+Fina, injected with testosterone undecanoate (125 mg/kg) and finasteride (0.8 mg/kg) after castration.
